# Albuminuria Reduction after High Dose of Vitamin D in Patients with Type 1 Diabetes Mellitus: A Pilot Study

**DOI:** 10.3389/fendo.2017.00199

**Published:** 2017-08-14

**Authors:** João Soares Felício, Alana Ferreira de Oliveira, Amanda Soares Peixoto, Ana Carolina Contente Braga de Souza, João Felício Abrahão Neto, Franciane Trindade Cunha de Melo, Carolina Tavares Carvalho, Manuela Nascimento de Lemos, Sávio Diego Nascimento Cavalcante, Fabricio de Souza Resende, Márcia Costa dos Santos, Ana Regina Motta, Luísa Corrêa Janaú, Elizabeth Sumi Yamada, Karem Miléo Felício

**Affiliations:** ^1^University Hospital João de Barros Barreto, Federal University of Pará, Endocrinology Division, Belém, Brazil

**Keywords:** vitamin D supplementation, diabetes kidney disease, type 1 diabetes mellitus, nephropathy, cholecalciferol, high dose vitamin D supplementation

## Abstract

**Background:**

Some studies suggest an association between diabetic kidney disease (DKD) and vitamin D (VD), but there is no data about the effect of high dose of VD on DKD in type 1 diabetes mellitus (T1DM). Our pilot study aims to evaluate albuminuria reduction in patients with T1DM supplemented with high dose of VD.

**Methods:**

22 patients received doses of 4,000 and 10,000 IU/day of cholecalciferol for 12 weeks according to patient’s previous VD levels. They were submitted to continuous glucose monitoring system, 24 hours ambulatory blood pressure monitoring and urine albumin-to-creatinine ratio before and after VD supplementation.

**Results:**

There was a reduction of DKD prevalence at the end of the study (68 vs 32%; *p* = 0.05), with no changes on insulin doses, glycated hemoglobin, glycemic variability and blood pressure values. A correlation between percentage variation of VD levels (ΔVD) and albuminuria at the end of the study was presented (*r* = −0.5; *p* < 0.05). Among T1DM patients with DKD at the beginning of the study, 8/13 (62%) had their DKD stage improved, while the other five ones (38%) showed no changes (*p* < 0.05).

**Conclusion:**

Our pilot study suggests an association between VD high dose supplementation, lower prevalence and improvement in stages of DKD in T1DM.

## Introduction

It is well known that better glycemic, blood pressure (BP) control and angiotensin-converting enzyme inhibitors (ACEIs) or angiotensin II receptor blockers (ARBs) are useful tools in early stages of diabetic kidney disease (DKD). Even though it remains an important cause of mortality in type 1 diabetes mellitus (T1DM) patients (1), vitamin D (VD) acts on renin–angiotensin system, inflammatory cascade, and vasculature (2, 3). In this scenario, it could be beneficial in prevention and progression of DKD.

There are other reports in literature investigating VD supplementation in DKD patients; however, they have been done with type 2 diabetes mellitus (T2DM) population. These studies have shown a significant improvement of serum HDL level with 6 months high-dose parenteral VD therapy (4) and a decrease in proteinuria (5). Although Derakhshanian et al. (6), in their meta-analysis, showed no significant change in proteinuria after supplementation with VD, but they agreed that risk for DKD is higher in VD-deficient patients with diabetes.

As we are aware, there is only one study with VD analog (paracalcitol) in T1DM, including 48 patients, that referred improvement in residual albuminuria (7). Nevertheless, the possible benefits of VD supplementation (cholecalciferol) in these cases are still not established. Our pilot study aims to evaluate possible albuminuria reduction in patients with T1DM and DKD, supplemented with high dose of VD.

## Materials and Methods

### Study Design

It is a 3-month prospective study, between January of 2015 and November of 2016, which aimed to evaluate albuminuria in patients receiving VD supplementation. Patients with insufficiency and/or VD deficiency [serum 25(OH)D less than 30 ng/mL] have received 10,000 IU/day for three consecutive months. Those with 25(OH)D levels between 30 and 60 ng/mL were treated with 4,000 IU/day of VD, in order to maintain serum levels above 30 ng/mL and less than 100 ng/mL. They were supplemented with cholecalciferol (1 mL = 20 drops = 4,000 IU). American Endocrine Society (8) has established values of 30 ng/mL as normal levels and the Institute of Medicine has used 20 ng/mL (9), so it remains controversial (10). We have done a supplementation of VD based only on basal VD levels to decide the dose, trying to achieve and maintain levels above 30 ng/mL. Even there is no consensus about normal VD levels, it would be ethically unappropriated not to replace VD in patients with deficiency or insufficiency, so our ethics committee has not approved a placebo group in this case.

Twenty-two individuals with T1DM were recruited from Endocrinology Division of the Federal University of Pará. The study was approved by ethics committee, reference number 0122.0.071.000-12, and it was in accordance with the standards of the National Health Council. The consent obtained was written and informed for all patients included in the study. Main inclusion criteria consisted in (a) T1DM patients with at least a 2-year follow-up; (b) age between 18 and 50 years in regular treatment with an endocrinologist; (c) body mass index (BMI) ≥18.5 kg/m^2^ and <40 kg/m^2^; (d) glycated hemoglobin (HbA1c) ≥7%; (e) no renal impairment [glomerular filtration rate (GFR) >89 mL/min/1.73 m^2^] (11); and (f) ACEI or ARB, hypertensive medications, basal, and ultra-rapid insulin must be in a stable dose for at least 3 months before starting the study. Exclusion criteria were (a) liver diseases; (b) bone metabolism diseases and previous VD supplementation; (c) pregnant women; (d) anemias; and (e) not controlled hypo- or hyperthyroidism and patients allergic to VD supplementation components.

Patients had to be in a stable dose of insulin for at least 3 months before starting the study. Basal insulin, ultra-rapid, regular, and metformin doses should be unchanged during the study. It is very important to point out that the inevitable changes in insulin dose were made only by usual care endocrinologist or patient by himself, never by study team. Patients were guided to inform the study team if an adverse event and/or recurrent hypoglycemia (capillary blood glucose <70 mg/dL), and/or hyperglycemia (blood glucose in capillary fasting ≥240 mg/dL in two consecutive days) occurred.

### Data Collection

Body mass index, BP, heart rate; laboratorial analysis [VD, urine albumin-to-creatinine ratio (UACR), HbA1c and GFR], 24 hours-ambulatory blood pressure monitoring (24h-ABPM) and continuous glucose monitoring system (CGMS) were performed in initial and final visit. HbA1c was analyzed by high-performance liquid chromatography, 25(OH)D by immunoassay (12); UACR was performed by immunoturbidimetry (Wiener-CMD800iX1) (13); GFR was calculated by the formula chronic kidney disease (CKD)-EPI (14). For purposes of data analysis, DKD was classified according to results in normoalbuminuria (<30 mg/g creatinine), microalbuminuria (≥30 mg/g creatinine and <300 mg/g creatinine), and macroalbuminuria (≥300 mg/g creatinine). Three first morning void urine sample collections were obtained before starting the study. The first morning void was defined as the subject’s first void after 5 a.m. Urine samples were collected in three different days, 3 weeks before visit one, to establish UACR. In three patients, it was not possible to collect three samples to UACR at the end of the study. Blood and urine samples were stored in a biorepository to future analysis for 10 years.

24-h ABPM was performed by the oscillometric method, installed in the morning and withdrawn after 24 h, and patient was instructed to maintain his usual activities and write them down in a diary, which should include the time and description of each activity performed. The device was programmed to perform a measurement every 15 min, and the arithmetic mean of systolic and diastolic BP was established for each hour, during the waking period, during sleep, and at 24 h.

Continuous glucose monitoring system sensor was introduced in regions with adequate fat layer (abdominal region was chosen in all patients in this study), with a needle device that was removed, remaining only the sensor in the subcutaneous and a transmitter attached externally to the skin. A monitor that was attached to patient clothing received the information by radio signal. Subcutaneous glucose was measured continuously in the form of an electrical signal. To calibrate the sensor, it was necessary for patient to verify the blood glucose at specific times (2, 6, and 12 h after installation, and every 12 h in subsequent days). The value obtained from capillary blood glucose was added to display. Patients have stayed 3–5 days with CGMS. About 864 glycemic values were measured into this period. The glycemic variability (GV) was evaluated by CGMS taking into account all glycemias measured during all days in each patient and standard deviation of glucose (SDG) was calculated by CGMS and used to evaluate GV.

### Statistical Analysis

Categorical variables were described as frequency (percentage). Numeric variables with normal distribution were described as mean (SD) and non-normally distributed as median (minimum–maximum). Chi-square, Fisher, and McNemar tests were used to compare categorical variables. The Student’s *t*-test and Mann–Whitney test were used to compare subgroups with and without normal distributions, respectively. The paired Student’s *t-*test and Wilcoxon test were used to compare data before and after intervention. To establish correlations between variables, Pearson and Spearman tests were used. The analysis of variance compared more than two subgroups with normal distribution, and the Kruskal–Wallis test was used to compare more than two subgroups without normal distribution.

In regard to DKD, the stages normoalbuminuria, microalbuminuria, and macroalbuminuria were used as DKD index 0, 1, and 2, respectively. For clarification, the index was used for statistical analysis. GV was assessed by SD of glycemic values. The percentage variations of some variables were calculated: total insulin dose percentage variation (Δ total insulin dose), basal insulin dose percentage variation (Δ basal insulin dose) percentage variation of prandial insulin dose (Δ prandial insulin dose), percentage variation of VD levels (ΔVD), and percentage variation in the standard deviation of glucose (Δ SDG).

Interferences are represented by hypothesis tests with a bilaterally significance level of 0.05. All information was stored and processed with the software SigmaStat (Jandel Scientific) version 3.5 and SPSS (Statistical Package for Social Sciences) 21.0 (IBM).

## Results

During the study, patients have not contacted the investigators to report or request care for serious and non-serious adverse event or side effects due to use of VD. The individual insulin doses were managed by usual care doctor or patient by himself. There was no change in metformin dose. In 22 patients, CGMS evaluated about 41,000 glycemias for SD analysis. In addition, approximately 2,000 BP measurements were performed using 24h-ABPM.

Clinical and laboratorial features at baseline and final of study are described in Tables 1 and 2, respectively. As expected, there was an increase in VD levels. A reduction of DKD prevalence at the end of the study (68 vs 32%; *p* = 0.05), with no changes on insulin doses, HbA1c, GV, and BP values occurred (Tables 2 and 3).

**Table 1:** Clinical characteristics of T1DM patients.

Variables	Initial (*N* = 22)	Final (*N* = 22)	*p*
Sex (M/F)	11/11	–	–
Age (years)	29.0 ± 8.1	–	–
Duration of T1DM (years)	11.3 ± 7.2	–	–
Office systolic BP (mmHg)	113.4 ± 14.9	112.3 ± 13.3	NS
Office diastolic BP (mmHg)	70.5 ± 11.5	69.4 ± 11.8	NS
BMI (kg/m^2^)	25.7 ± 3.4	25.6 ± 3.5	NS

*T1DM, type 1 diabetes mellitus; BP, blood pressure; BMI, body mass index; NS, not significant*.

**Table 2:** Laboratorial characteristics and ABPM of type 1 diabetes mellitus patients.

Variables	Initial (*N* = 22)	Final (*N* = 22)	*p*
Glycated hemoglobin (%)	9.3 ± 2.7	9.3 ± 2.3	NS
SDG	62.5 (31–96)	66.5 (33–111)	NS
Vitamin D (ng/mL)	25.9 (14.2–53.9)	36.8 (9.4–122.5)	<0.01
Creatinine (mg/dL)	0.94 ± 0.15	0.98 ± 0.18	NS
Glomerular filtration rate (mL/min/1.73 m^2^)	108.6 ± 23.2	106.5 ± 24.1	NS
Albuminuria (log_10_mg/24 h)	1.72 ± 0.51	1.65 ± 0.37	NS
Albuminuria index	0.75 ± 0.55	0.68 ± 0.48	NS
DKD prevalence (yes/no); *N* = 19	13/6 (68%)	6/13 (32%)	0.05
Vigil systolic blood pressure (BP) (mmHg)	120.1 ± 10.3	120.9 ± 13.0	NS
Vigil diastolic BP (mmHg)	74.9 ± 9.0	76.8 ± 10.5	NS
Sleep systolic BP (mmHg)	110.0 ± 11.6	110.9 ± 12.7	NS
Sleep diastolic BP (mmHg)	67.0 ± 10.0	68.3 ± 10.9	NS
SD of sleep systolic BP (mmHg)	8.2 ± 3.3	10.3 ± 4.9	NS
SD of sleep diastolic BP (mmHg)	8.0 ± 2.2	8.7 ± 2.7	NS

**Table 3:** Insulin doses before and after vitamin D dose supplementation.

Variables	Initial (*N* = 22)	Final (*N* = 22)	*p*
Basal insulin (IU)	44.2 ± 20.1	42.6 ± 19.2	NS
Prandial insulin (IU)	22.0 ± 12.6	20.8 ± 11.9	NS
Total insulin (IU)	65.2 ± 29.9	62.4 ± 27.1	NS

*Total insulin = basal + prandial insulin (IU)*.

*NS, not significant*.

**Figure 1:**
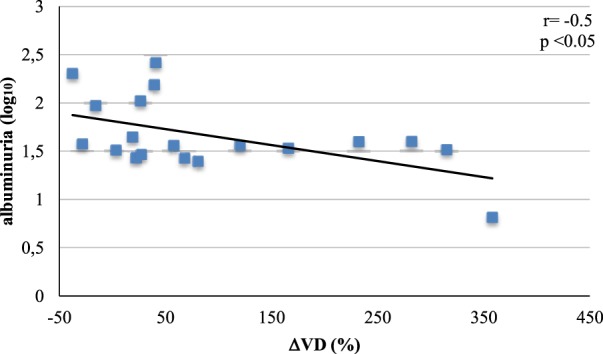
Correlation between albuminuria at the end of study and percentage variation (Δ) of vitamin D (VD).

A correlation between percentage variation of VD levels (ΔVD) and albuminuria at the end of study was presented (*r* = −0.5; *p* < 0.05) (Figure 1). There was no change in mean insulin doses (basal and prandial) before and after VD supplementation (Table 3).

In addition, in one patient, it was not possible to perform 24h-ABPM at the end of the study.

**Figure 2:**
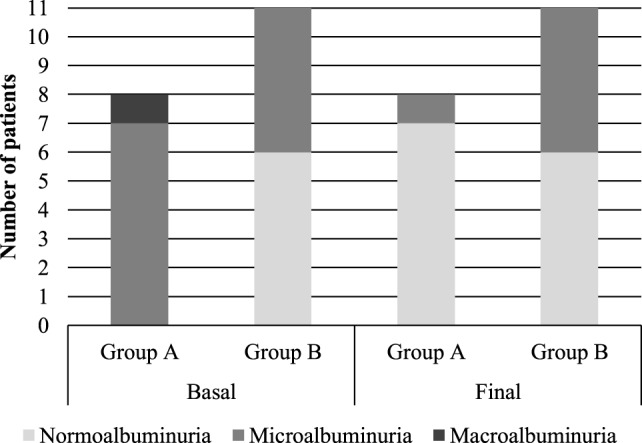
Diabetic kidney disease stages before and after vitamin D supplementation.

Only in 19 patients, it was possible to establish the stage of DKD based on three UACR samples at the end of the study. To understand the changes in DKD stages, patients were divided into two groups: those in whom there was an improvement in the stage of DKD (Group A, *n* = 8) and those who have no changes in DKD stage (Group B, *n* = 11). No patient presented worsening in the stage of DKD. In group A, seven patients have improved from stage of microalbuminuria to normoalbuminuria and one patient from macroalbuminuria to microalbuminuria, showing regression of DKD. In group B, there was no DKD stage change in any patient, all of them remained with microalbuminuria or normoalbuminuria at the end of the study (*p* = 0.01) (Figure 2).

## Discussion

Our pilot study suggests an association between VD supplementation and an improvement in DKD stages on patients with T1DM. We have also found a correlation between improvement in percentage VD levels and reduction of albuminuria.

Recently, there was just one study (7) in the literature that supplemented 48 patients with T1DM and DKD for 12 weeks with a VD analog (paracalcitol). They found a significant reduction in the rate of urinary albumin excretion in these patients. However, this study has not selected patients in early DKD stages and has not used high dose supplementation, as we did. Therefore, even though we have a lower sample, as we are aware, our pilot study is the first to demonstrate an improvement in DKD stages, after high dose of VD (cholecalciferol) supplementation, in T1DM patients.

In the same way, VITAL study (15) in 2010 found an improvement in residual albuminuria, using 2 µg of paricalcitol (VD analog) for supplementation in patients with T2DM and DKD. By contrast, a reduction has not been demonstrated in individuals using 1 µg daily, as well as those on placebo. Therefore, it remains controversial the optimum level of VD supplementation in DKD and if there is any possible benefit of this therapeutic strategy in disease progression.

A few non-interventional studies have demonstrated an association between VD and DKD, opening the door to investigate if VD supplementation can bring benefits to patients with this complication. Verrotti et al. (16), comparing VD levels and urinary albumin excretion in 22 patients with T1DM and microalbuminuria, 24 with normoalbuminuria, and 24 controls, showed lower levels of VD in the first group. De Boer et al. (17) in 2012, have also found that low plasma concentrations of VD (below 20 ng/mL) were associated with an increased risk of microalbuminuria in 65% of patients with T1DM. In addition, recently, we have compared T1DM subjects with controls and observed a progressive decline in VD levels/status as DKD worsened. Through linear regression analysis, we have suggested that a possible association between higher VD levels and lower values of microalbuminuria was independent of HbA1c and BP levels, hypothesizing a kidney direct effect of VD (18). Nonetheless, it is necessary to clarify how VD supplementation could bring improvements on this scenario.

In 2015, Derakhshanian et al. (6), in a meta-analysis, evaluated four interventional studies that used VD (calcitriol or cholecalciferol) in patients with T2DM to reduce proteinuria. The largest one (19), with 91 patients, showed proteinuria reduction. The other three studies (20–22) have failed to reduce urinary protein excretion. All of them have included patients with several stages of CKD. In our study, all patients were T1DM, had normal GFR—so, they were on early stages of DKD—and had taken a much higher VD dose. Therefore, these studies cannot fit as a comparison parameter to our protocol. We need to continue recruiting to increase our sample and reinforce our data.

In relation to the mechanisms that VD could bring some benefits to DKD management, some studies suggest that VD could inhibit the renin–angiotensin system, a possible mediator of progressive renal injury in DKD (17, 23–25). Other possible hypotheses would be alterations in the nitric oxide and reduction of the inflammation and fibrosis, which would play a critical role in the development of this complication, by promoting endothelial dysfunction, with increased vascular permeability and release of pro-inflammatory molecules, which over time would lead to progression of renal injury and alterations in BP (26–29).

Vitamin D acts directly on beta cells facilitating insulin secretion from the binding of 1,25(OH)2D3 to its nuclear VD receptor and indirectly by regulating the flow of calcium in those cells (30, 31). Changes in the concentrations of this mineral can lead to peripheral resistance to insulin action, by reduction of signal transduction in glucose transport activity. In addition, hypovitaminosis D contributes to the increase of chronic low-grade inflammation associated with insulin resistance, contributing to beta cell apoptosis (32, 33).

The main limitation of our study was the sample size that needs to be enlarged. However, as we have used high supplementation dose of VD, it was necessary to evaluate preliminary results of our pilot study to verify possible benefits that allow us to increase the recruitment. In addition, study design is also a limitation, considering that it is a not randomized controlled study.

In summary, our pilot study suggests an association between VD high dose supplementation and albuminuria reduction in patients with TDM1 and DKD.

## Availability of Data and Material

The datasets during and/or analyzed during the current study available from the corresponding author on reasonable request.

## Ethics Statement

Research ethics committee of João de Barros Barreto Hospita. Informed consent was obtained from all patients for being included in the study. All procedures followed were in accordance with the ethical standards of the responsible committee on human experimentation (institutional and national) and with the Helsinki Declaration of 1975, as revised in 2008.

## Author Contributions

All persons who meet authorship criteria are listed as authors, and all authors certify that they have participated sufficiently in the work to take public responsibility for the content, including participation in the concept, design, analysis, writing, or revision of the manuscript. JF and KF took part in conception and design of study. EY, MS, AS, and FM were responsible for acquisition of data, while SC, LJ, CC, and JN have done the analysis and interpretation of data. ML, AO, AP, and FR have drafted the manuscript together. All authors have revised the manuscript critically and approved the version to be published.

## Conflict of Interest Statement

The authors declare that the research was conducted in the absence of any commercial or financial relationships that could be construed as a potential conflict of interest.

## Abbreviations

T1DM, type 1 diabetes mellitus; T2DM, type 2 diabetes mellitus; VD, vitamin D; DKD, Diabetic Kidney Disease; HbA1c, glycated hemoglobin; Δ SDG, Standard Deviation of Glucose; GV, glycemic variability; GC, glycemic control; CGMS, continuous glucose monitoring system; 24h-ABPM, 24 hours ambulatory blood pressure monitoring; GFR, glomerular filtration rate; HR, heart rate; UACR, urine albumin-to-creatinine ratio; BMI, body mass index; BP, blood pressure; HPLC, high-performance liquid chromatography; ADA, American Diabetes Association; DCCT, Diabetes Control And Complications Trial Research Group; VDR, VD receptor; VDRE, VD responsive element; ACEIs, angiotensin-converting enzyme inhibitors; ARBs, angiotensin II receptor blockers.
